# Genetic determinants for the racial disparities in the risk of prostate and testicular cancers

**DOI:** 10.1038/s43856-022-00205-5

**Published:** 2022-11-02

**Authors:** Ivie Uzamere, Yinqiao Wang, Tongzhang Zheng, Yong Zhu

**Affiliations:** 1 Department of Environmental Health Sciences, Yale University School of Public Health, New Haven, CT 06520 USA; 2 Department of Epidemiology, Brown University School of Public Health, Providence, RI 02912 USA

**Keywords:** Predictive markers, Cancer epidemiology

## Abstract

**Background:**

A worldwide higher incidence of prostate cancer and lower incidence of testicular cancer in men of African ancestry compared to European ancestry has been observed previously. However, underlying mechanisms accounting for these observations are largely unknown.

**Methods:**

The current study analyzed previously reported SNPs associated with either prostate cancer or testicular cancer to examine whether the risk allele frequency could help us understand the observed incidence disparities in men of African ancestry and European ancestry. Both t-test and regression analysis were performed.

**Results:**

Here we show that men of African ancestry are more likely to have risk alleles of prostate cancer and less likely to have risk alleles of testicular cancer compared to men of European ancestry.

**Conclusions:**

Our findings suggest that genetic factors may play an important role in the racial disparities in the risk of prostate and testicular cancers.

## Plain language summary

It has been observed that men of African ancestry have a higher incidence of prostate cancer and lower incidence of testicular cancer compared to men of European ancestry. However, little is known about underlying mechanisms accounting for these observations. The current study compares frequencies of all genetic alterations associated with risks of prostate cancer or testicular cancer between the two racial groups. Our findings suggest that differences in the frequencies of genetic alterations between the groups may help to explain the racial disparities in the risk of prostate and testicular cancers.

## Abstract (web summary)

Uzamere, Wang et al. seek to understand the genetic determinants of racial disparities in the risk of prostate and testicular cancers. The authors identify differences in average risk allele frequency for known prostate/testicular cancer-associated variants between men of African descent and men of European descent.

## Introduction

Prostate cancer (Pca) and testicular cancer, also known as testicular germ cell tumor (TGCT), are common cancers diagnosed in men within the United States and globally^1,2^. The patterns of incidence between these two cancer types differ greatly between men of different geographical origin, age group, race and ethnicity^1^. In regard to race and ethnicity, interestingly, prostate cancer and testicular cancer display opposite trends in incidence in men of European ancestry and African ancestry. Studies have shown that men of African ancestry have disproportionately higher incidence and mortality rates compared with men of European ancestry in prostate cancer^3^. Whereas men of European ancestry are more likely to be diagnosed and have higher rates of mortality than men of African ancestry in testicular cancer^4^. However, little is known about underlying mechanisms accounting for these observations.

Genetic factors play a major role in cancer etiology. In the past decade, many cancer-related genetic variants such as single nucleotide polymorphisms (SNPs) have been identified in genetic association studies, especially genome-wide association studies (GWAS). The current study analyzed previously reported SNPs associated with either prostate cancer or testicular cancer to examine whether the risk allele frequency could help us understand the observed incidence disparities in men of African ancestry and European ancestry. Our results show that men of African ancestry are more likely to have risk alleles of prostate cancer and less likely to have risk alleles of testicular cancer compared to men of European ancestry, which suggest that genetic factors may play an important role in the racial disparities in the risk of prostate and testicular cancers.

## Methods

### Data collection

Literature search was performed by using combinations of key words including, single nucleotide polymorphism (SNP), Genome Wide Associate Study (GWAS), genetic risk variant, prostate cancer, and testicular cancer. Genome-wide association studies and meta-analysis of genome wide association studies were used to collect all SNPs associated with testicular cancer and prostate cancer. SNPs associated with these two cancer types at an adjusted statistically significant level (p < 0.05) were collected. Risk allele frequencies of these identified SNPs in populations of African and European ancestry were then obtained from 3 databases: 1000 Genomes Project, Allele Frequency Aggregator (ALFA), and the genome Aggregation Database (gnomAD). The 1000 Genomes Project database contains data for 2,504 individuals from 26 populations^5^. The ALFA database has over 2 million subjects from 12 diverse populations^6^. GnomAD has 125,748 exomes and 15,708 genomes from unrelated individuals sequenced, totaling 141,456 individuals^7^. The number of individuals with available allele frequency data for each SNP in these 3 databases were included in Supplementary Data 1, 2.

### Calculation of allele frequency

Allele frequencies in different databases may vary slightly and the frequency of each risk allele (F) was calculated by averaging frequencies in these 3 databases with justification for sample sizes. To further account for attributable risk of the risk alleles, weighted frequency (F_w_) was calculated using the equation: F_w_ = F*OR/OR_max_, where OR is the corresponding odds ratio of the risk allele and OR_max_ is the largest odds ratio among risk alleles.

### Statistics

Average weighted frequencies of risk alleles were then compared between the 2 racial/ethnic groups using a one-tailed student’s t-test because the testing hypotheses have one direction of interest for each cancer type. The t-test was conducted by using Graphpad Prism (version 8.2.1). Deming regression analysis was performed using Graphpad Prism (version 8.2.1) to find the line of best fit by accounting for frequency variations on both the x- and the y- axis. Regression lines from both cancer types were compared to a standard regression line that has equal allele frequencies in both African and European populations (slope = 1). Data source for these analyses were included in Supplementary Data 3, 4.

To further consider the potential effect of SNPs in linkage disequilibrium (LD) on our analyses, we interrogated LD correlations for SNPs located on the same chromosome using a web-based tool (https://ldlink.nci.nih.gov) that uses subjects from all available ethnic groups and draws data from the 1000 Genomes Project. *r*^2^ > 0.8 was used as the threshold to determine LD and all linked SNPs were indicated in Supplementary Data 1, 2. Average allele frequencies of the SNPs in LD were calculated and used in analysis. However, we did not combine allele frequencies of linked SNPs if (1) they are located in different protein coding genes, (2) they are located in functional regions (e.g., 3′-UTR), and (3) their frequencies between risk and reference alleles show opposite directions (risk allele frequency is higher in one ethnic group and lower in another).

### Reporting summary

Further information on research design is available in the Nature Research Reporting Summary linked to this article.

## Results and discussion

A total of 226 risk SNPs significantly associated with prostate cancer and 79 risk SNPs significantly associated with testicular cancer were identified (Supplementary Data 1, 2). Most SNPs included in our analysis were obtained from GWAS, replication studies, and meta-analysis studies. Two SNPs with conflicting results from two different studies were excluded from further analysis. Data of risk and reference alleles, odds ratio (OR), confidence interval (CI), adjusted p-value, gene symbol, ethnic group, number of cases and controls, and citation of original publications were collected and included in supplementary data files. When there were multiple ORs present in previous studies, we only presented the ones from meta-analysis studies. When a SNP was investigated in multiple ethnicity groups, we chose to present the result that showed the most significant OR. While some SNPs were identified and verified in multiple races/ethnicities, most study subjects are individuals of European ancestry. Due to the low power of small sample sizes, differences in allele frequencies, or different linkage disequilibrium patterns, non-European populations in many previous studies did not show significant associations.

**Fig. 1:**
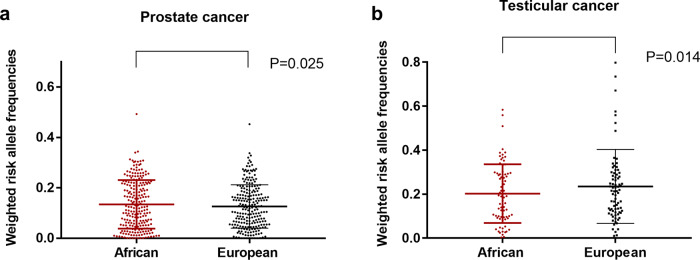
Comparison of risk allele frequencies of prostate and testicular cancers between men of African descent and European descent. **a** Average weighted risk allele frequency of prostate cancer was significantly higher in men of African descent compared to men of European ancestry descent (*n* = 226; *p* = 0.025). **b** Average weighted risk allele frequency of testicular cancer was significantly lower in men of African descent compared to men of European descent (*n* = 79; *p* = 0.014).

The hypothesis that a higher/lower incidence of prostate/testicular cancer could be associated with a higher/lower average frequency of risk alleles was tested by comparing risk allele frequencies between European and African populations. For prostate cancer, the average risk allele frequencies (F) was significantly higher in men of African ancestry (45%) compared to men of European ancestry (42%) (n = 226; *p* = 0.045). After considering the odds ratio of risk alleles, the analysis of weighted frequencies (F_w_) stayed highly significant (*n* = 226; *p* = 0.025) (Fig. 1a). On the contrary, the average risk allele frequencies of testicular cancer was significantly lower in men of African ancestry (46%) compared to men of European ancestry (51%) (n = 79; *p* = 0.030). The weighted analysis showed the same significant difference (*n* = 79; *p* = 0.014) (Fig. 1b).

**Fig. 2:**
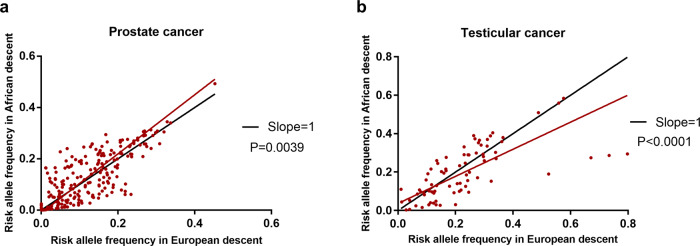
Regression lines of risk allele frequencies between African and European descent for prostate and testicular cancers. **a** The regression line of prostate cancer data (slope = 1.152) skewed above the standard line (slope = 1) towards African population. The differences between the slopes were highly significant (*n* = 226; *p* = 0.0039). **b** The regression line of testicular cancer data (slope = 0.705) skewed below the standard line slope (slope = 1) towards European population. The differences between the slopes were highly significant (*n* = 79; *p* < 0.0001).

Moreover, frequencies of all risk alleles in African (Y axis of Fig. 2) and European (X axis of Fig. 2) populations showed significant linear regression relationships in both prostate cancer (Y = 1.152*X-0.011, *p* = 0.0039) and testicular cancer (Y = 0.705*X + 0.0367, *p* < 0.0001). The differences between the slopes were highly significant where the regression line of prostate cancer data (slope = 1.152, *p* = 0.0039, Fig. 2a) skewed above the standard line towards the African population and the regression line of testicular cancer data (slope = 0.705, *p* < 0.0001, Fig. 2b) skewed below the standard line slope towards the European population.

To further analyze if a particular locus or outlier affected our results, we removed certain data points that could potentially skew the relationships. For the secondary prostate cancer analysis, we removed 31 SNPs located at the chromosome 8q24 locus because multiple studies have suggested that variants in the 8q24 region were significantly associated with prostate cancer. After the removal, differences in risk allele frequencies between African and European ancestry were insignificant, suggesting that genetic variants at the chromosome 8q24 locus are major contributors to the racial difference of prostate cancer incidence.

Similarly, we removed 4 outliers (rs995030, rs1508595, rs4474514, rs3782179) that skewed down the regression line and found no significant difference between European and African risk allele frequencies of testicular cancer (*p*-value = 0.157), suggesting that these 4 SNPs contribute the most to the deviation of the slope. Interestingly, 3 of the 4 SNPs (rs995030, rs4474514, rs3782179) are located in the *KITLG* gene, which is found by previous studies to be involved in the development of TGCTs and presents a strong specific risk factor independently from spermatogenic function^8^. However, while *KITLG* might play a role in TGCT development, few studies investigated the testicular cancer risk allele frequency differences between individuals of African and European ancestries, except for *KITLG*’s significant associations with pigmentation of hair, eye and skin between Africans and Europeans^9,10^. Thus, potential functional roles of *KITLG* in testicular cancer, especially between different racial groups, warrant further investigation.

After consideration of SNPs in LD, results from the t-test remained significant for both prostate cancer (*p*-value = 0.019) and testicular cancer (*p*-value=0.048). We also conducted the analyses without considering SNP locations (in potential functional regions). We found that the t-test result for prostate cancer remained significant (*p* = 0.036) and the t-test for testicular cancer became insignificant (*p* = 0.142). For the testicular cancer analysis, SNPs in LD are all located in the *KITLG* gene. After taking average allele frequencies of these SNPs, the number of data points was reduced, and the t-test result was affected. However, this finding provides further evidence of the importance of the *KITLG* gene in testicular cancer etiology.

Results from both t-test and regression analysis are consistent with each other, which indicate that men of African ancestry are more likely to have risk alleles of prostate cancer and less likely to have risk alleles of testicular cancer compared to men of European ancestry. These findings suggest that genetic factors could partially explain the greater burden of prostate cancer on men of African ancestry and the higher incidence of testicular in men of European ancestry compared to other racial/ethnic groups. Our findings are consistent with results from a newly published work on prostate cancer, which compared allele frequencies of 269 prostate cancer loci and the distribution of polygenic risks scores (PRS) for prostate cancer between African and European men^11^.

Cancer is a complex disease that normally involves multiple genes and environmental factors in its etiology. Therefore, in addition to understanding the influence of genetic factors, social and environmental factors also need to be considered when analyzing the observed racial and ethnic disparities in the risk of prostate and testicular cancers.

We realize that SNPs detected from association studies may be a proxy of the functional ones in the region. It is important to compare frequency data of functional SNPs between human populations for testing our hypothesis. However, this approach is not feasible for the current study due to limited information available for the functional SNPs in the LD chromosomal regions and the lack of functional data available for many SNPs identified from association studies. Future mechanistic investigations on genetic variants in the identified genes will facilitate the selection of SNPs for such comparisons between different racial groups.

## Supplementary information

Description of Additional Supplementary Files

Supplementary Data 1

Supplementary Data 2

Supplementary Data 3

Supplementary Data 4

Reporting Summary

## Data Availability

All data generated or analyzed during this study are from literature search and information on selected SNPs are obtained from 3 public databases (the 1000 Genomes Project, ALFA, and gnomAD). Details of all data used in the study are included in the Supplementary Data files 1, 2. Source data used to generate Figs. 1 and 2 are included in the Supplementary Data files 3 and 4.
