# Supplementary material for: Genetic determinants for the racial disparities in the risk of prostate and testicular cancers
Source: Commun Med (Lond). 2022 Nov 2;2:138. doi: 10.1038/s43856-022-00205-5 (PMC9630379; doi:10.1038/s43856-022-00205-5)
Supplement: Supplementary file 1 — Description of Additional Supplementary Files [file 43856_2022_205_MOESM1_ESM.pdf]

Description of Additional Supplementary Files

File Name: Supplementary Data 1

Description: Risk Allele Frequencies of Prostate Cancer. The data table shows risk allele frequencies obtained from 3 available databases (1000 Genome, ALFA and gnomAD), weighted risk allele frequencies by Odds Ratio (OR), and other SNP information (accession number, gene symbol, chromosomal location) from the original publications.

File Name: Supplementary Data 2

Description: Risk Allele Frequencies of Testicular Cancer. The data table shows risk allele frequencies obtained from 3 available databases (1000 Genome, ALFA and gnomAD), weighted risk allele frequencies by Odds Ratio (OR), and other SNP information (accession number, gene symbol, chromosomal location) from the original publications.

File Name: Supplementary Data 3

Description: Weighted Risk Allele Frequencies of Prostate Cancer. These source data are used to generated Figure 1a and 2a.

File Name: Supplementary Data 4

Description: Weighted Risk Allele Frequencies of Testicular Cancer. These source data are used to generated Figure 1b and 2b.
